# Comparison of the inverted internal limiting membrane flap technique without versus with an autologous blood clot for treating macular hole-associated retinal detachment

**DOI:** 10.1186/s40662-024-00417-x

**Published:** 2025-01-02

**Authors:** Ke Zhu, Yingchao Wang, Boya Lei, Ling Chen, Yanqiong Zhang, Qing Chang, Gezhi Xu, Yingqin Ni

**Affiliations:** 1 Eye Institute and Department of Ophthalmology, Eye and Ear Nose, Throat Hospital of Fudan University, 83 Fen Yang Road, Shanghai, 200031 China; 2 Shanghai Key Laboratory of Visual Impairment and Restoration, Fudan University, Shanghai, China; 3 Key Laboratory of Myopia and Related Eye Diseases, NHC, Key Laboratory of Myopia and Related Eye Diseases, Chinese Academy of Medical Sciences, Shanghai, China

**Keywords:** Macular hole-associated retinal detachment, Internal limiting membrane flap technique, Autologous blood clot, Macular hole closure, Best-corrected visual acuity

## Abstract

**Background:**

To investigate the anatomical and functional outcomes of macular hole-associated retinal detachment (MHRD) after vitrectomy using the inverted internal limiting membrane (ILM) flap technique with autologous blood clot (ABC).

**Methods:**

This retrospective observational study included 80 eyes with MHRD that underwent vitrectomy with ILM flap without (46 eyes) or with ABC (34 eyes). Fundus photography and optical coherence tomography were evaluated. The pre- and postoperative best-corrected visual acuities (BCVAs) and BCVA improvement were compared between the two groups.

**Results:**

The MH closure rates after initial surgery were similar in the ILM flap group and ILM flap with ABC group [40 (87%) vs. 29 (85%) eyes, respectively]. The proportion of eyes with hyperreflective bridging tissue (HBT) was lower in the ILM flap group than ILM flap with ABC group [13 (32%) vs. 16 (55%) eyes, *P* = 0.060]. The postoperative improvement in BCVA was significantly better in the ILM flap group (*P* = 0.027). Multiple linear regression analysis revealed that preoperative BCVA was positively associated with postoperative improvement in BCVA (β = 0.638, *P* = 0.000), while the ILM flap with ABC technique was negatively associated with postoperative improvement in BCVA (β =  − 0.299, *P* = 0.039, adjusted r^2^ = 0.415).

**Conclusions:**

The inverted ILM flap technique alone resulted in better foveal configurations and visual outcomes than the ILM flap technique combined with ABC in patients with MHRD.

## Background

Macular hole-associated retinal detachment (MHRD) is one of the most vision-threatening ocular disorders in highly myopic eyes and is highly prevalent in East Asia [1–4]. The pathogenesis of MHRD may be related to tangential traction from the posterior hyaloid membrane and epiretinal membrane [5, 6], inverse traction from the anatomical mismatch between the neurosensory retina and retinal pigment epithelium (RPE)–choroid–sclera complex that occurs during ocular axis extension [4, 7]. Chorioretinal atrophy may also weaken retinal adherence [4, 8, 9].

Since pars plana vitrectomy (PPV) with gas tamponade was first reported in 1982 [10], several surgical procedures, including PPV with silicone oil (SO) tamponade, macular buckling, scleral imbrication, and removal of the epiretinal membrane and internal limiting membrane (ILM) have been introduced to treat MHRD [5, 7, 9, 11]. Although ILM peeling can remove the tangential traction [5], it cannot compensate for the anatomical mismatch caused by posterior staphyloma [6]. The retinal reattachment rate ranged from 85.1% to 100% [1, 2, 5, 12–14], but the macular hole (MH) closure rate remained unsatisfactory. The inverted ILM flap technique was recently developed to treat MHs with or without retinal detachment (RD) [4, 14–16]. An ILM flap covering the MH acts not only as a filler but also as a scaffold to correct the anatomical mismatch between the neurosensory retina and the posterior staphyloma [4, 15]. Compared with conventional ILM peeling, the ILM flap technique improved the MH closure rate, the anatomical foveal configuration, and the postoperative visual outcomes [4, 14–16]. The ILM flap technique is highly demanding and there is a risk of flap dislocation after surgery [17]. Blood and its derivatives have recently been explored as an adjuvant to act mechanically as a glue to enhance adhesion of the ILM in eyes with large or refractory MHs [17–21]. In addition to the cellular components of whole blood, such as platelets, various serum factors were shown to promote retinal healing [17, 18, 20, 22]. The inverted ILM flap technique combined with an autologous blood clot (ABC) has been used to treat large or refractory MHs [17–19, 21]. However, few studies have investigated the efficacy of this technique in patients with MHRD. Therefore, we performed a retrospective observational study to compare the anatomical and functional outcomes of the inverted ILM flap technique with or without an ABC for treating MHRD.

## Methods

### Patients

We reviewed the medical records of patients with MHRD who underwent PPV with the inverted ILM flap technique with or without application of an ABC at the Eye and ENT Hospital of Fudan University (Shanghai, China) between March 2017 and August 2023. The study was approved by the Institutional Review Committee of the Eye and ENT Hospital of Fudan University (Reference No. 2024191) and was conducted in accordance with the ethical standards stated in the Declaration of Helsinki. All treatments and ophthalmological examinations were explained to the patients, who then provided informed consent.

The study included Chinese patients aged 18–75 years who had been diagnosed with MHRD [RD extending by more than 1 disk diameter (DD) around the margin of the MH] [8, 23, 24]. Patients were excluded if they had a history of RD or other vitreoretinal diseases, preexisting ocular diseases, or a history of ocular trauma or surgery other than refractive or cataract surgery.

The baseline demographics and ocular characteristics, including type of posterior staphyloma, presence of macular retinoschisis, subtype of MHRD, surgical procedures, and surgical outcomes, were collected from the medical records. The types of posterior staphyloma were determined according to the study by Ohno-Matsui and Jonas [25]. MHRD was classified based on the extension of RD, as described by Lai et al. [18]. Type I was defined as RD within the vascular arcade and type II as RD beyond the arcade. Color fundus photography (IMAGEnet R, Topcon, Tokyo, Japan) and ultra-widefield fundus imaging (California, Optos, Dunfermline, UK) were performed. Macular horizontal, vertical sectional, and volume scans were obtained by optical coherence tomography (OCT; Spectralis OCT, Heidelberg Engineering, Heidelberg, Germany).

### Surgical procedures

All procedures were performed by experienced surgeons (YN, GX, YZ, LC, and TZ). Standard 23-gauge, three-port PPV was performed under retrobulbar or general anesthesia. Phakic eyes underwent phacoemulsification before vitrectomy if fundus visibility was obscured by the lens. After removing the central core vitreous, the posterior vitreous cortex was identified with 0.1 mL/2 mg of triamcinolone acetonide aqueous suspension (40 mg/1 mL suspension, Kunming Jida Pharmaceutical Company, Yunnan, China). ILM peeling was facilitated by staining with 0.1 mL/0.1 mg of indocyanine green (25 mg dry indocyanine green dye diluted in 5% glucose water, Dandong Yichuang Pharmaceutical Company, Liaoning, China). The ILM, with a size of 1.0–1.5 DD, was preserved around the MH and the residual ILM was circumferentially peeled to the margins of the vascular arcade. The ILM was not completely detached from the retinal surface and remained anchored at the margin of the MH, as described by Shin et al. [26]. After air–fluid exchange, the subretinal fluid (SRF) was gently drained from the MH. Perfluorocarbon liquid (RT DECALIN, Carl Zeiss Meditec AG, Jena, Germany) was intravitreally injected to stabilize the ILM. The lifted ILM flap was massaged and gently inverted to cover the entire MH using forceps. If the SRF was drained through an intentional hole in the peripheral retina, perfluorocarbon liquid was injected to stabilize the ILM and the lifted ILM flap was inverted. After air–fluid exchange, the SRF was drained from the intentional hole.

**Fig. 1:**
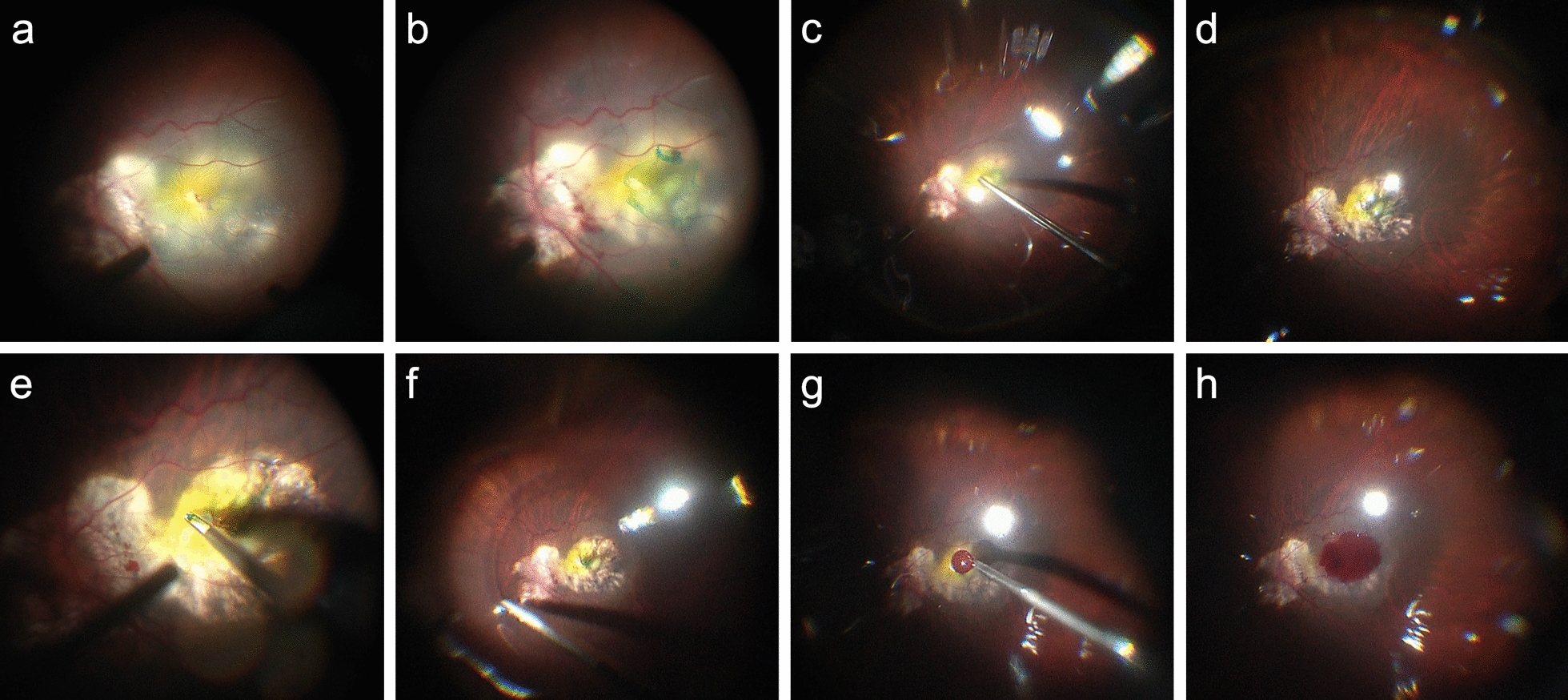
Images showing the surgical procedure of pars planar vitrectomy combined with the inverted internal limiting membrane (ILM) flap technique and an autologous blood clot (ABC) for repairing macular hole-associated retinal detachment in highly myopic eyes. **a** After removing the posterior vitreous cortex, ILM peeling was facilitated by staining with 0.1 mL/0.1 mg of indocyanine green. **b** The ILM, with a size of 1.0–1.5 disk diameters, was preserved around the MH and the residual ILM was circumferentially peeled to the margins of vascular arcade. The ILM was not completely detached from the retinal surface and remained anchored at the margin of the MH. **c** After air–fluid exchange, the subretinal fluid was gently drained from the MH. **d** The retina was well attached. **e** Perfluorocarbon liquid was injected intravitreally to stabilize the ILM. The lifted ILM flap was massaged and gently inverted to cover the entire MH using forceps. **f** The perfluorocarbon liquid was removed. **g** After completing the ILM technique, one drop of fresh blood obtained from the patient under sterile conditions was gently injected onto the surface of the MH. **h** The fresh blood clotted on the macular surface, and the ILM flap and ABC formed a macular plug that sealed the MH within a few minutes

After completing the ILM technique, autologous blood was applied to a consecutive series of patients to evaluate its clinical application value. Approximately 1 mL of fresh blood was obtained from the patient’s dorsal hand vein under sterile conditions. One drop of fresh blood was injected gently onto the surface of the MH. The fresh blood clotted on the macular surface, and the ILM flap and ABC mixture quickly formed a macular plug that sealed the MH within several minutes (Fig. 1). The peripheral retina was carefully checked to detect any retinal tears or lattice degeneration where laser photocoagulation was applied. The surgeon confirmed that the ILM flap remained in place and performed perfluoropropane gas [15% C3F8 (ISPAN, Alcon Laboratories Inc., Texas, USA)] or SO (5000–5900 mPas, Oxane 5700, Bausch & Lomb Inc., New York, USA) tamponade at the end of surgery. Gas tamponade was performed when the retinal detachment was within the vascular arcade and the proliferative vitreoretinopathy was grade A or B. The patients were asked to stay in a prone position for ≥ 8 h per day for 2 weeks postoperatively. SO was removed ≥ 3 months after surgery or earlier in patients with uncontrolled intraocular pressure.

### Postoperative evaluation of anatomical and functional outcomes

**Fig. 2:**
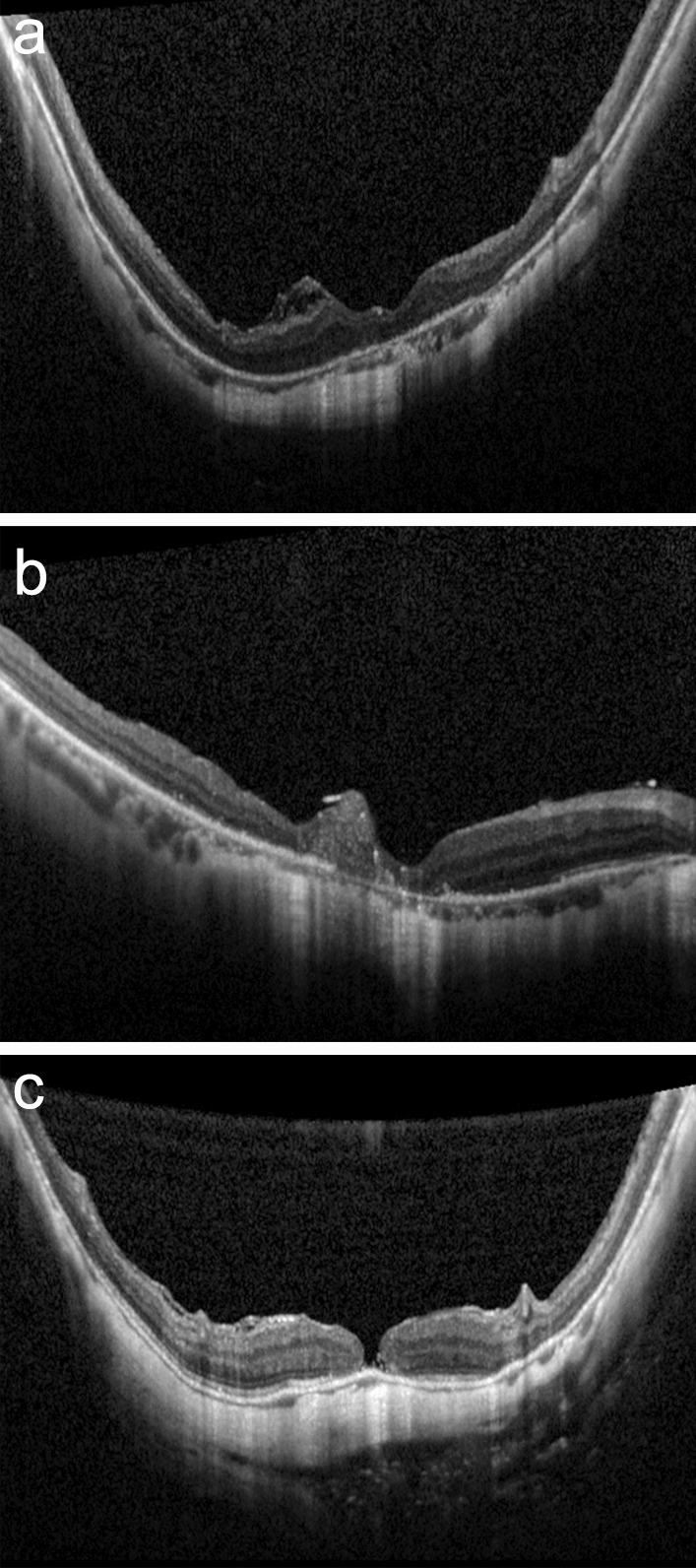
Representative optical coherence tomography images demonstrating the patterns of foveal configuration in eyes with closure of the macular hole (MH). **a** A closed MH with normal foveal contour with mild to moderate backscattering of layers, which formed a smooth circular surface to cover the retinal pigment epithelium (RPE). **b** Hyperreflective bridging tissue appeared in the fovea and sealed the MH. **c** An unclosed MH with foveal defects of the neurosensory retina, flat edges, and a bare RPE

Retinal reattachment was defined as complete absorption of the SRF and full attachment of the neurosensory retina to the RPE. MH closure was defined as the absence of neurosensory defects over the fovea on OCT images (Fig. 2) [17]. The central foveal thickness was manually measured using the “measure distance” function in the OCT software in 69 eyes. We also evaluated the presence of hyperreflective bridging tissue (HBT) and the foveal microstructures, including recovery of the outer nuclear layer (ONL), external limiting membrane (ELM), and ellipsoid zone (EZ) [14]. Two researchers (KZ and YW) assessed the patterns of MH closure and the integrity of the reflection line showing the foveal microstructures on OCT images obtained at the final follow-up, and consensus was reached for each patient. The ratio of the maximum height to the minimum diameter of the unclosed MH was calculated as the traction hole index (THI), as described by Ruiz-Moreno et al. [27]. The postoperative BCVA, and its improvement at the final follow-up, were assessed as functional outcomes. The minimum follow-up duration was 6 months, with an average follow-up duration of 12.39 months.

### Statistical analyses

The BCVA in Snellen values was converted to the logarithm of the minimum angle of resolution (logMAR) for statistical analysis. Counting fingers (CF) and hand movements were assigned the equivalent logMAR of 2.0 and 3.0, respectively. SPSS statistical analysis software (Version 22.0, SPSS Inc., Chicago, USA) was used for all statistical analyses. Differences in baseline demographics, ocular characteristics, surgical procedures, and surgical outcomes were compared using the t test or χ^2^ test. Fisher’s exact test was used if the expected number of patients was less than five. Linear regression analysis was used to identify associations between clinical factors and postoperative improvement in BCVA. *P* values of less than 0.05 indicated statistically significant differences.

## Results

We reviewed the medical records for a total of 80 eyes of 20 males and 59 females. The baseline demographics, ocular characteristics, and surgical procedures are shown in Table 1. All 80 eyes underwent 23-gauge, three-port PPV; 44 eyes underwent PPV combined with cataract surgery, and 16 eyes underwent cataract surgery after vitrectomy. Fifty-eight eyes (72%) underwent SO tamponade, and 22 eyes (28%) underwent perfluoropropane (C_3_F_8_) tamponade. SO was removed from all 58 eyes. The surgeons performed both techniques, with 46 eyes undergoing PPV with the inverted ILM flap technique (ILM flap group) and 34 eyes undergoing the inverted ILM flap technique combined with an ABC (ILM flap with ABC group).

**Table 1:** Comparison of baseline clinical factors and surgical procedures

Characteristics N (%) or Mean ± SD	Total (n = 80)	ILM flap group (n = 46)	ILM flap with ABC group (n = 34)	*P* value
Demographics				
Sex, female	59 (74)	32 (70)	27 (80)	0.322
Age (years)	61 ± 9	60 ± 8	62 ± 10	0.565
Ocular characteristics				
Duration of decreased vision (months)	11.31 ± 30.43	9.66 ± 36.47	13.55 ± 19.86	0.575
Preoperative BCVA (logMAR)	1.87 ± 0.69	1.99 ± 0.64	1.70 ± 0.74	0.069
Preoperative BCVA (Snellen)	20/1483	FC	20/1002	
Axial length (mm)	29.15 ± 2.56	28.73 ± 2.84	29.72 ± 2.07	0.090
Staphyloma type				0.024
Type I, wide, macular	34 (43)	17 (37)	17 (50)	
Type II, narrow, macular	37 (46)	21 (46)	16 (47)	
Type III, peripapillary	1 (1)	0 (0)	1 (3)	
Macular retinoschisis	54 (68)	34 (74)	20 (59)	0.154
MHRD type				0.502
Type I, within arcade	25 (31)	13 (28)	12 (35)	
Type II, beyond arcade	55 (69)	33 (72)	22 (65)	
Lens status, phakia/pseudophakia	63 (79)/17 (21)	36 (78)/10 (22)	27 (79)/7 (21)	0.901
Surgical procedures				
Combined cataract surgery, PEA + IOL or PEA only	44 (55)	22 (48)	22 (65)	0.134

*ABC* = autologous blood clot; *BCVA* = best-corrected visual acuity; *FC* = finger counting; *ILM* = internal limiting membrane; *IOL* = intraocular lens; *logMAR* = logarithms of the minimum angle of resolution; *PEA* = phacoemulsification and aspiration; *SD* = standard deviation

### Surgical outcomes

The surgical outcomes of the two groups are compared in Table 2. No infection or inflammation was detected in any eye. Retinal reattachment was achieved after initial surgery in 43 eyes (93%) in the ILM flap group and in 32 eyes (94%) in the ILM flap with ABC group. Four eyes required a second surgery, and one eye required a third surgery to achieve final reattachment. The MH was closed after initial surgery in 40 eyes (87%) in the ILM flap group, which was comparable with the rate in the ILM flap with ABC group [29 eyes (85%)]. The mean BCVA (logMAR) improved significantly from 1.99 ± 0.62 (CF) preoperatively to 1.24 ± 0.54 (20/348) in the ILM flap group at the final follow-up examination (*P* < 0.001), and from 1.70 ± 0.74 (20/1002) to 1.28 ± 0.63 (20/381) in the ILM flap with ABC group (*P* < 0.001). The improvement in BCVA was significantly better in the ILM flap group than in the ILM flap with ABC group (*P* = 0.027).

**Table 2:** Comparison of postoperative outcomes

Characteristics N (%) or Mean ± SD	ILM flap group (n = 46)	ILM flap with ABC group (n = 34)	*P* value
MH closure	40 (87)	29 (85)	> 0.999
Postoperative BCVA (logMAR)	1.24 ± 0.54	1.28 ± 0.63	0.765
Postoperative BCVA (Snellen VA ratio)	20/348	20/381	
BCVA improvement (logMAR)	0.74 ± 0.71	0.42 ± 0.52	0.027
BCVA improvement by ≥ 0.3 logMAR	36 (78)	22 (65)	0.180
Follow-up period (months)	12.74 ± 10.25 (6.00 to 50.00)	11.91 ± 6.33 (6.00 to 26.00)	0.679

*ABC* = autologous blood clot; *BCVA* = best-corrected visual acuity; *ILM* = internal limiting membrane; *logMAR* = logarithms of the minimum angle of resolution; *MH* = macular hole; *SD* = standard deviation; *VA* = visual acuity

The pattern of foveal configuration and the recovery of foveal microstructures in 69 eyes with MH closure are compared between the two groups in Table 3. The proportion of eyes with HBT was lower in the ILM flap group than in the ILM flap with ABC group [13 eyes (32%] vs. 16 eyes (55%), *P* = 0.060]. There were no differences in the proportions of eyes with ONL (*P* > 0.999), ELM (*P* = 0.634), or EZ (*P* > 0.999) recovery between the two groups. The THI of the unclosed MH was 1.49 ± 0.26 in the ILM flap group and 1.48 ± 0.18 in the ILM flap with ABC group (*P* = 0.985).

**Table 3:** Comparison of postoperative MH recovery

Foveal microstructure recovery of the closed MH N (%) or Mean ± SD	ILM flap group (n = 40)	ILM flap with ABC group (n = 29)	*P* value
CFT (μm)	168.10 ± 136.64	146.67 ± 107.61	0.486
Foveal configuration			
U type closure	24 (60)	13 (45)	0.212
V type closure	3 (8)	0 (0)	0.258
HBT	13 (32)	16 (55)	0.060
Foveal microstructure			
ONL recovery	1 (3)	0 (0)	> 0.999
ELM recovery	3 (8)	1 (3)	0.634
EZ recovery	1 (3)	0 (0)	> 0.999

*ABC* = autologous blood clot; *CFT* = central foveal thickness; *ELM* = external limiting membrane; *EZ* = ellipsoid zone; *HBT* = hyperreflective bridging tissue; *ILM* = internal limiting membrane; *MH* = macular hole; *ONL* = outer nuclear layer

### Associations of clinical factors and surgical procedures with visual outcomes

Univariate linear regression analysis revealed that preoperative BCVA [regression coefficient β = 0.598, 95% confidence interval (CI): 0.434 to 0.762, *P* = 0.000, r^2^ = 0.403] and SO tamponade (β = 0.360, 95% CI: 0.042 to 0.677, *P* = 0.027, r^2^ = 0.061) were positively associated with the improvement in BCVA. By comparison, the ILM flap with ABC technique was negatively associated with the improvement in BCVA (β =  − 0.324, 95% CI: − 0.611 to − 0.038, *P* = 0.027, r^2^ = 0.061). After adjusting for confounding variables, multiple linear regression analysis revealed that only preoperative BCVA (β = 0.638, 95% CI: 0.456 to 0.820, *P* = 0.000) was positively associated and the inverted ILM flap with ABC technique (β =  − 0.299, 95% CI:  − 0.582 to − 0.016, *P* = 0.039) was negatively associated with the improvement in BCVA (Table 4).

**Table 4:** Multiple linear regression of factors associated with the postoperative BCVA improvement in MHRD

Variables Included in the Model	Univariate		Multivariable	
	β (95% CI)	*P* value	β (95% CI)	*P* value
Demographic and ocular characteristics				
Sex, female	− 0.169 (− 0.499 to 0.161)	0.311		
Age (years)	0.008 (− 0.009 to 0.024)	0.367		
Duration of decreased vision (months)	− 0.002 (− 0.007 to 0.003)	0.431		
Preoperative BCVA (logMAR)	0.598 (0.434 to 0.762)	0.000	0.638 (0.456 to 0.820)	0.000
Axial length (mm)	− 0.026 (− 0.083 to 0.030)	0.357		
High myopia	− 0.336 (− 0.817 to 0.146)	0.170		
Type II posterior staphyloma	− 0.050 (− 0.353 to 0.253)	0.743		
Retinoschisis	− 0.007 (− 0.320 to 0.305)	0.963		
Retinal detachment beyond arcade	0.051 (− 0.264 to 0.366)	0.749		
Lens status, phakia	− 0.145 (− 0.501 to 0.211)	0.420		
Surgical procedures				
Combined cataract surgery	0.032 (− 0.262 to 0.326)	0.830		
Tamponade agent, SO	0.360 (0.042 to 0.677)	0.027	− 0.284 (− 0.632 to 0.058)	0.101
ILM flap with ABC technique	− 0.324 (− 0.611 to − 0.038)	0.027	− 0.299 (− 0.582 to − 0.016)	0.039
Surgical outcomes				
Initial retinal reattachment	0.330 (− 0.270 to 0.930)	0.276		
MH closure	0.204 (− 0.218 to 0.626)	0.339		
U or V type closure	0.167 (− 0.124 to 0.457)	0.257		
HBT	− 0.131 (− 0.449 to 0.187)	0.412		
CFT (μm)	0.000 (− 0.001 to 0.002)	0.464		
ONL recovery	0.883 (− 0.418 to 2.185)	0.181		
ELM recovery	0.643 (− 0.014 to 1.299)	0.055		
EZ recovery	0.857 (− 0.447 to 2.160)	0.194		
Follow-up period (months)	0.000 (− 0.017 to 0.017)	0.971		

Adjusted *r*^2^ = 0.415

*ABC* = autologous blood clot; *β* = regression coefficient; *BCVA* = best-corrected visual acuity; *CFT* = central foveal thickness; *CI* = confidence interval; *ELM* = external limiting membrane; *EZ* = ellipsoid zone; *HBT* = hyperreflective bridging tissue; *ILM* = internal limiting membrane; *logMAR* = logarithms of the minimum angle of resolution; *MH* = macular hole; *MHRD* = macular hole-associated retinal detachment; *ONL* = outer nuclear layer; *SO* = silicone oil

## Discussion

In myopic eyes, MHRD is one of the most severe disorders that threaten vision [1–4, 18]. Michalewska et al. [15, 16] introduced the inverted ILM flap technique to facilitate MH closure and improve visual outcomes in large MHs. Kuriyama et al. [4] extended the technique to the treatment of MHRD. Using the inverted ILM flap technique, the MH closure rate increased to 75%–100% [4, 14, 28]. Blood components have recently been explored as an adjuvant to achieve better MH outcomes in eyes with large or refractory MHs [17–21]. Lai et al. [18] developed a novel approach using ILM flap technique combined with ABC and reported MH closure rates of 96% when treating MHRD. These reports hypothesized that an ABC could not only act mechanically as a glue to enhance adhesion of the ILM and reduce the risk of postoperative flap dislocation, but also act physiologically as a potential autologous adjuvant to augment healing processes [17–20, 22]. However, no studies have compared the anatomical or functional outcomes of the ILM flap technique without or with an ABC in patients with MHRD. Therefore, our study was performed to address this gap.

**Fig. 3:**
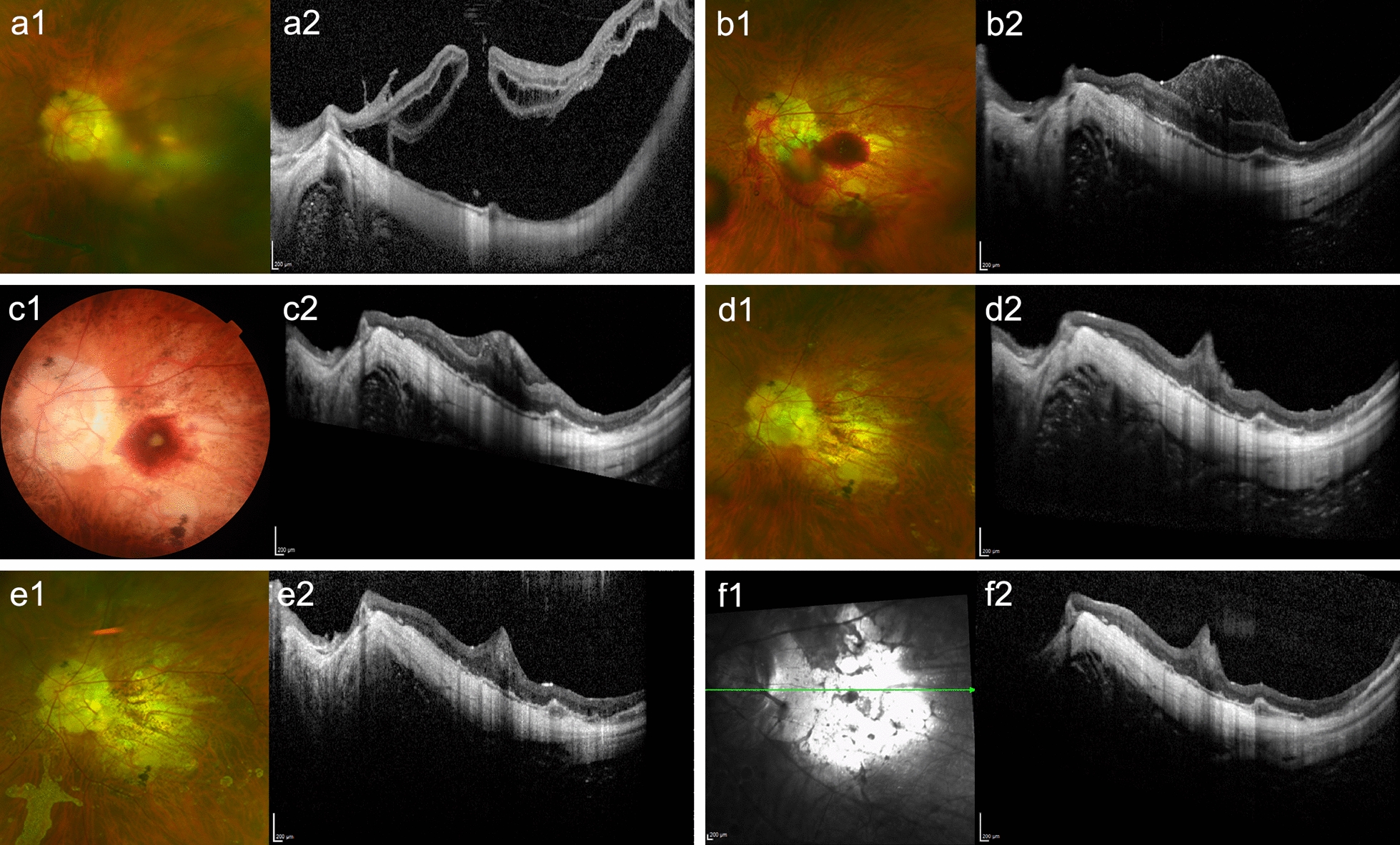
Representative ultra-widefield fundus photography and optical coherence tomography (OCT) images demonstrating the postoperative follow-up of a patient who underwent the inverted internal limiting membrane (ILM) flap technique with an autologous blood clot. **a1, a2** Ultra-widefield fundus photography and OCT revealed type II macular hole (MH)-associated retinal detachment before surgery. The visual acuity was counting fingers at 30 cm. **b1** One day after the surgery, the blood clot is layered over the inverted ILM flap to form a macular plug closing the MH. **b2** The retina was well attached. **c1** The blood clot persisted for 1 month after surgery. **c2** The ILM flap and blood clot complex sealed the MH. **d1** The blood clot was completely absorbed 2 months after surgery. **d2** The MH was closed, and hyperreflective bridging tissue (HBT) appeared at the fovea. **e1**, **e2** Four months after surgery, the size of HBT was stable and a defect in the outer retinal layer was visible. **f1**, **f2** Eight months after surgery, the HBT remained, and the outer retinal integrity did not recover. The patient’s visual acuity at the final follow-up examination remained counting fingers at 30 cm

The inverted ILM flap technique improved MH closure in patients with MHRD [4, 14–16]. ILM mechanically compensates for the shortening of the retina in the posterior pole [29], serves as a scaffold for astrocytes and Müller cell gliosis [15], provides activated Müller cells and neurotrophic factors to encourage MH healing [30], and facilitates glial cell proliferation to result in a favorable foveal configuration [31]. MH closure was achieved in 69 eyes (86%) in our study. Postoperative OCT revealed that the rate of MH closure with HBT was greater in the ILM flap with ABC group than in the ILM flap group, with borderline statistical significance. The slightly elevated hyperreflective tissue representing the macular plug after surgery with blood application was observed in several studies [18, 21]. A flat flap may promote MH closure with a relatively normal foveal contour [4, 14–16, 21]. However, blood application might have a negative effect [18, 20]. The underlying mechanism may involve the following: autologous blood contains various growth factors (GFs), such as vascular endothelial GF, platelet-derived GF, and epidermal GF, which facilitate tissue regeneration [17, 20, 22]. These GFs activate multiple transduction signal pathways when in contact with disintegrated neuroretinal tissue [22] to promote MH healing. They increase proliferation, differentiation, and migration of retinal glial cells and RPE cells [20, 22], which are crucial for retinal repair, angiogenesis, and regeneration [17, 20, 22]. The trophic factors and GFs also coordinate various communication pathways, including the fibrin formation network, to restore retinal integrity, and thus provide a provisional extracellular matrix for harboring retinal cells [32]. ILM peeling shaves the basal membrane of Müller cells, thereby acting as a stimulus for proliferation [19]. The ILM flap provides sufficient fibrosis and induces prolonged foveal glial proliferation [4, 14–16, 21]. After blood application, the ILM flap and ABC mixture form a macular plug that completely seals the entire MH (Fig. 3). The GFs in autologous blood further facilitate glial and fibroblast proliferation with the help of ILM acting as a bridge [18, 20, 21]. Excessive gliosis, induced by these synergistic effects, could harm retinal neurons and promote fibrotic tissue formation [20, 21], which may impair reconstitution of the foveal microstructures and hinder functional recovery [18, 21]. Furthermore, the excessive gliosis that fills the MH may prevent reconnections among the retinal layers and affect the recovery of microvascular blood flow [21]. In turn, inadequate microvascular blood flow may further hinder restoration of the foveal microstructure [21]. Moreover, the migration of blood into the subretinal space may release free radicals and proinflammatory substances that affect the regeneration of retinal neurons [33]. Given that the use of the inverted ILM flap alone already provides good stability, ABC could be more beneficial for recurrent MHRD in which an intrinsic instability of a free ILM flap or an autologous retinal transplantation flap may require the adjunction of ABC.

MH closure is pivotal to the final visual outcome in MHRD [4, 14–16]. In this study, the high anatomical success rate also resulted in favorable postoperative functional recovery, and vision was improved or remained stable in 70 eyes (86%) at the final follow-up examination. The ILM flap may act as a bridge for naked cones [28], facilitate the regeneration of Müller cells [28], and create a microenvironment conducive to the repositioning of photoreceptors in direct proximity with the fovea [15], all of which contribute to visual improvement [34]. Here, we compared the visual outcomes of the ILM flap technique with or without ABC for MHRD. The postoperative improvement in BCVA was significantly better in the ILM flap group than in the ILM flap with ABC group. Multiple regression analysis revealed that the preoperative BCVA was positively associated but the inverted ILM flap technique with ABC was negatively associated with the postoperative improvement in BCVA. There are some possible explanations for this. First, combining the inverted ILM flap technique with blood application likely stimulated gliosis through a synergistic process more strongly than the ILM flap technique alone [18, 20, 21]. The increased risk of excessive gliosis could have a detrimental effect by impairing reconstitution of the foveal microstructures and create unfavorable conditions for photoreceptor repositioning [21, 35], hindering further visual recovery [18]. The contraction of excessive glial tissue may cause centripetal movement of photoreceptors [19]. The excessive glial tissue may also increase the risk of cytotoxic damage to retinal ganglion cells [36]. Second, the leakage of blood into the subretinal space may have a toxic effect on photoreceptors due to the release of free radicals and proinflammatory substances that hamper the regeneration of retinal neurons [33]. Third, the persistence of the macular plug [18], which mainly consists of excessive glial tissue, may cause central visual disturbances, scotomas, or distorted vision [14]. In addition, the patients in the ILM flap with ABC group complained of central visual occlusion that lasted for more than 1 month, until the blood clot was completely resolved. Therefore, we think that the ABC not only leads to a smaller improvement in BCVA in the long term but also has a short-term impact on postoperative visual quality.

Regarding the surgical procedure, other than applying blood as an adjuvant, the major modification in this study involved using an inverted ILM flap to cover the surface of the MH instead of inserting the ILM to fill the MH. This modification could prevent the instruments from touching the RPE and choroid, reducing the risk of severely damaging these vulnerable tissues in highly myopic eyes [18].

Several limitations of our study should be addressed. First, the follow-up period was probably too short to observe full recovery of the foveal microstructures. The functional recovery may occur gradually, over several years after MH surgery [20]. Second, this was a retrospective observational study, and the use of ABC was not randomly selected. Thirdly, it was difficult to evaluate the extent of myopic degeneration before the formation of MHRD, which may confound the visual outcomes of the two techniques. Finally, fresh blood is more visible and accessible to manipulate. Other potential blood components, such as platelets, could be explored in future studies.

## Conclusion

We found that the inverted ILM flap technique alone resulted in relatively better foveal configurations and greater improvements in BCVA compared with the inverted ILM flap technique combined with ABC when treating MHRD. The preoperative BCVA was positively associated and the inverted ILM flap with ABC was negatively correlated with postoperative visual outcomes.

## Data Availability

The datasets generated during and/or analyzed during the current study are available from the corresponding author upon reasonable request.

## Abbreviations

ABC: Autologous blood clot
BCVA: Best-corrected visual acuity
CF: Counting fingers
CFT: Central foveal thickness
CI: Confidence interval
DD: Disk diameter
ELM: External limiting membrane
ERM: Epiretinal membrane
EZ: Ellipsoid zone
GF: Growth factors
HBT: Hyperreflective bridging tissue
ILM: Internal limiting membrane
logMAR: Logarithms of the minimum angle of resolution
MH: Macular hole
MHRD: Macular hole-associated retinal detachment
OCT: Optical coherence tomography
OR: Odds ratio
PPV: Pars plana vitrectomy
RD: Retinal detachment
RPE: Retinal pigmented epithelium
SD: Standard deviation
SO: Silicone oil
SRF: Subretinal fluid
THI: Traction hole index
